# Perceived Facilitators of and Barriers to Implementation of a Decision Support Tool for Adolescent Depression and Suicidality Screening: Focus Group and Interview Study

**DOI:** 10.2196/26035

**Published:** 2021-09-15

**Authors:** Ana Radovic, Nathan Anderson, Megan Hamm, Brandie George-Milford, Carrie Fascetti, Stacey Engster, Oliver Lindhiem

**Affiliations:** 1 Department of Pediatrics, University of Pittsburgh School of Medicine UPMC Children's Hospital of Pittsburgh Pittsburgh, PA United States; 2 University of Pittsburgh School of Medicine Pittsburgh, PA United States; 3 Center for Research on Healthcare's Data Center University of Pittsburgh Pittsburgh, PA United States; 4 UPMC Western Psychiatric Hospital Pittsburgh, PA United States; 5 Clinical and Translational Science Institute Pediatric PittNet University of Pittsburgh Pittsburgh, PA United States; 6 Department of Psychiatry University of Pittsburgh Pittsburgh, PA United States

**Keywords:** depression, adolescent, suicidality, screening

## Abstract

**Background:**

Screening Wizard (SW) is a technology-based decision support tool aimed at guiding primary care providers (PCPs) to respond to depression and suicidality screens in adolescents. Separate screens assess adolescents’ and parents’ reports on mental health symptoms, treatment preferences, and potential treatment barriers. A detailed summary is provided to PCPs, also identifying adolescent-parent discrepancies. The goal of SW is to enhance decision-making to increase the utilization of evidence-based treatments.

**Objective:**

This qualitative study aims to describe multi-stakeholder perspectives of adolescents, parents, and providers to understand the potential barriers to the implementation of SW.

**Methods:**

We interviewed 11 parents and 11 adolescents and conducted two focus groups with 18 health care providers (PCPs, nurses, therapists, and staff) across 2 pediatric practices. Participants described previous experiences with screening for depression and were shown a mock-up of SW and asked for feedback. Interviews and focus groups were transcribed verbatim, and codebooks were inductively developed based on content. Transcripts were double coded, and disagreements were adjudicated to full agreement. Completed coding was used to produce thematic analyses of the interviews and focus groups.

**Results:**

We identified five main themes across the interviews and focus groups: parents, adolescents, and pediatric PCPs agree that depression screening should occur in pediatric primary care; there is concern that accurate self-disclosure does not always occur during depression screening; SW is viewed as a tool that could facilitate depression screening and that might encourage more honesty in screening responses; parents, adolescents, and providers do not want SW to replace mental health discussions with providers; and providers want to maintain autonomy in treatment decisions.

**Conclusions:**

We identified that providers, parents, and adolescents are all concerned with current screening practices, mainly regarding inaccurate self-disclosure. They recognized value in SW as a computerized tool that may elicit more honest responses and identify adolescent-parent discrepancies. Surprisingly, providers did not want the SW report to include treatment recommendations, and all groups did not want the SW report to replace conversations with the PCP about depression. Although SW was originally developed as a treatment decision algorithm, this qualitative study has led us to remove this component, and instead, SW focuses on aspects identified as most useful by all groups. We hope that this initial qualitative work will improve the future implementation of SW.

## Introduction

Adolescent suicide rates have increased by 20% in the past decade and are now the second leading cause of death for ages 10 to 24 years in the United States [1,2].

### Screening for Depression and Suicidality

As depression and suicidal ideation are strong risk factors for adolescent suicidal behavior [3], screening for depression and suicidality have become national priorities, with depression screening being a billable International Classification of Diseases, Tenth Revision, diagnosis code and a covered preventive service often used as a quality measure in pediatric quality initiatives [4]. The United States Preventive Services Task Force recommendations highlight that screening programs *alone* are unlikely to improve care for depression or have a measurable impact on reducing suicide rates among adolescents. In fact, despite routine screening in primary care settings, initiation of depression treatment following a positive screening has been as low as 17% [5,6].

### Barriers to Treatment Initiation Despite Screening

The reasons include primary care providers’ (PCPs) unfamiliarity and variability in the interpretation of screening results [7], failure to assess and address patients’ and parents’ barriers to treatment [8], failure to factor in patients’ and parents’ preferences [9,10], and low motivation for treatment among patients who screen positive for depression. The revised Guidelines for Adolescent Depression in Primary Care (GLAD-PC) recommends that PCPs assess and integrate information about patient beliefs, preferences, and barriers to guide their management decisions; however, further research is needed to implement these guidelines [11]. Although the adolescent age group may be defined as ages 12 to 26 years, in this manuscript, we refer to early and middle adolescence (ages 12-17 years), considering that earlier access to mental health treatment may improve long-term health outcomes.

### Shared Decision-making and Comorbidity Assessment to Address Barriers to Screening

**Figure 1:**
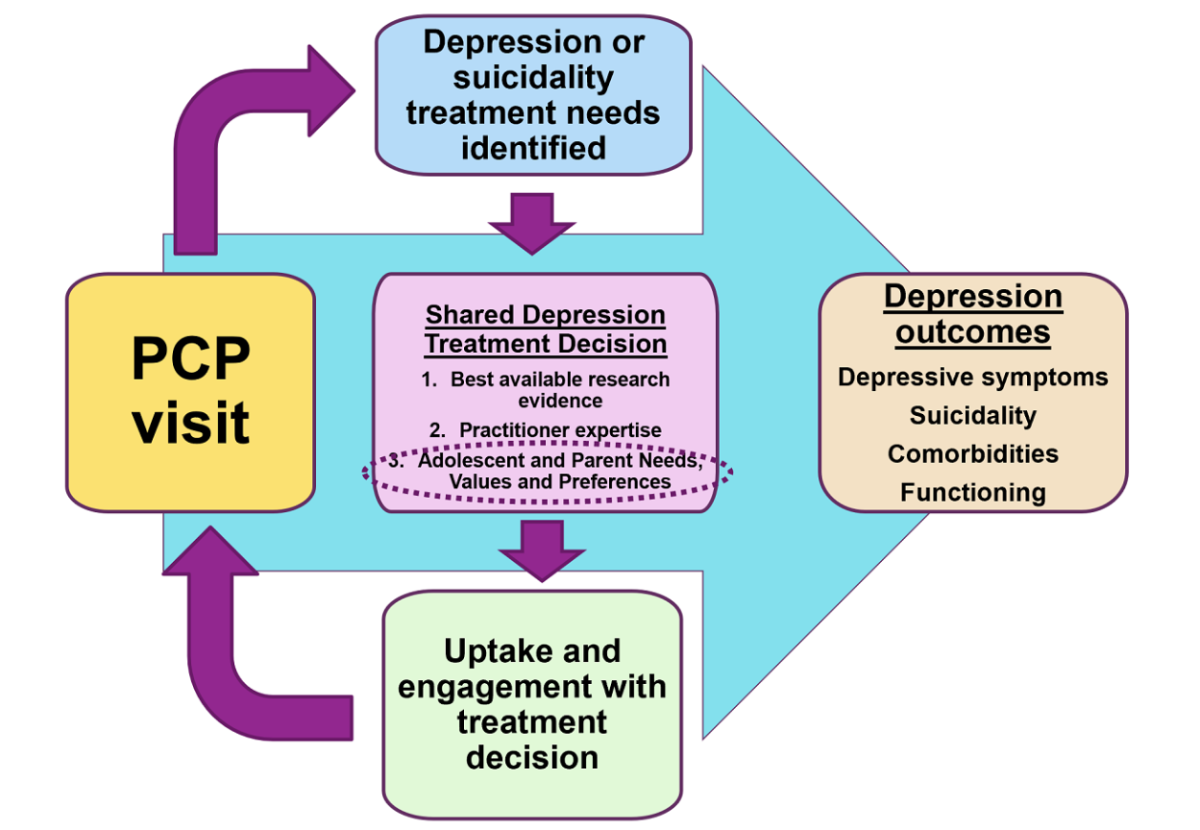
Primary care provider depression treatment decision-making. PCP: primary care provider.

As presented in Figure 1, identifying the need for depression treatment triggers a complex decision-making process. Optimally, this process incorporates a triad of perspectives to reach a treatment decision: (1) the PCPs’ clinical experience and knowledge of diagnostic and treatment evidence and (2) the adolescents’ and (3) the parents’ personal expertise in their values, beliefs, and preferences, which may or may not be aligned [12]. Although routine screens offer identification of depressive symptomatology, the PCP must also consider potential comorbidities and ask separately about suicide when making their management decision. Anxiety [13] and mania are important to consider, especially when considering antidepressant prescribing for depression, which may induce a switch to mania for those at risk for bipolar disorder [14]. Not asking about substance use may lead to treatment failure and is associated with a higher risk of suicide attempt [15]. Suicidality may be missed if not assessed independently. For example, when the short Patient Health Questionnaire (PHQ)-2 version of the PHQ-9 is asked, 20% of youth with suicidal ideation may be missed [16,17]. After formulating a diagnostic and management decision based on clinical data, the PCP will need to align their recommendations with patient preferences [18]. Although in some states, adolescents may confidentially consent for mental health care, a parent or guardian is typically involved in medical decision-making and provides the instrumental support needed to obtain treatment. Similarly, barriers to treatment are common and differ between adolescents and their parents [10], which PCPs may not be aware of [19], unless they elicit them. Shared decision-making (SDM) interventions that assess treatment preferences and barriers in the context of clinical decision-making tend to improve decisional quality by reducing conflict around decision-making [20,21] and have proven to be more successful than non-SDM interventions for depression and other chronic illnesses [22,23], as evidenced by systematic reviews conducted globally. SDM is recommended for PCPs assessing adolescent depression in the current GLAD-PC guidelines, specifically that “the patient and family should be active team members and approve the roles of the PCP and mental health clinicians” [11]. However, busy PCPs tend to engage in few SDM behaviors for depression [24], even when they possess knowledge and resources to recommend evidence-based care [25]. This process of eliciting, interpreting, and addressing the previously stated information is quite complex and may lead to prolonged visits for a busy PCP, especially those practicing in busy community pediatric primary care settings.

### Opportunities for Technology to Enhance Routine Depression Screening

Advances in technology that assist PCPs (eg, decision support tools) with interpreting screening results and then provide personalized treatment recommendations may improve treatment outcomes for mental health [26]. Specifically, in pediatric primary care, computerized screens with associated decision support have promoted provider utilization of depression screening results [27] and identification of and assessment for suicidality [28]. Most caregivers are comfortable with adolescents independently completing computerized screening [29]. Adolescents also find that computerized tools, especially those that provide personalized feedback, may enhance their interaction with their health care providers [30].

### The Screening Wizard Tool

The aim of this study is to develop a technology-based decision support tool, *Screening Wizard* (SW), aimed at guiding PCPs in responding to positive depression and suicidality screens in adolescents. SW was developed as an iPad (Apple Inc) app to include both an adolescent screen and a parent screen. Each screen would include a series of questions that would ask about adolescents’ mental health—including anxiety, mania, and suicidality comorbidities—and substance use symptoms and parents’ perception of their adolescent’s symptoms; adolescents’ mental health treatment preferences and readiness if treatment is recommended that day as well as the parent’s readiness and preferences on behalf of their child; and both adolescents’ and parents’ potential barriers to mental health treatment. The results of this screen and accompanying patient handouts (with referral information and psychoeducation) would then be summarized in a report that highlights adolescent-parent discrepancies to share with the health care provider. Our goal is to facilitate SDM between adolescents, parents, and PCPs and to increase uptake of depression treatment in the primary care setting.

### This Study

Despite its anticipated benefits, the potential challenges of integrating new technology interventions in busy community primary care settings may be numerous. This manuscript describes a multi-stakeholder qualitative study conducted with adolescents, parents, and providers to evaluate the usability of a prototype of the SW and understand the acceptability of and potential barriers to future implementation of SW.

## Methods

This qualitative study was conducted as part of a larger study meant to refine the already-developed SW technology in a prototype form before implementation.

### SW Prototype

**Figure 2:**
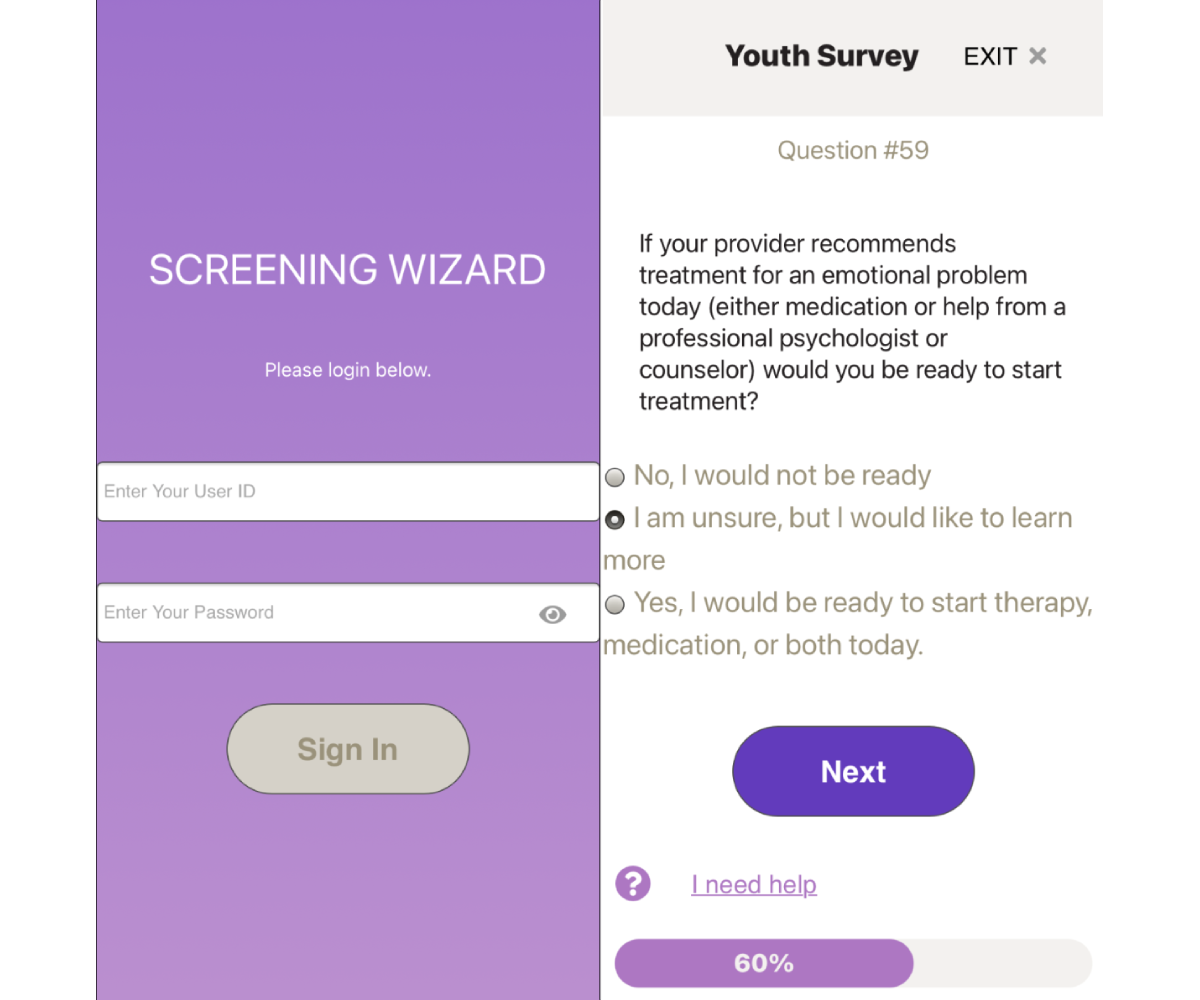
Example screenshots from the Screening Wizard interface for adolescents.

**Figure 3:**
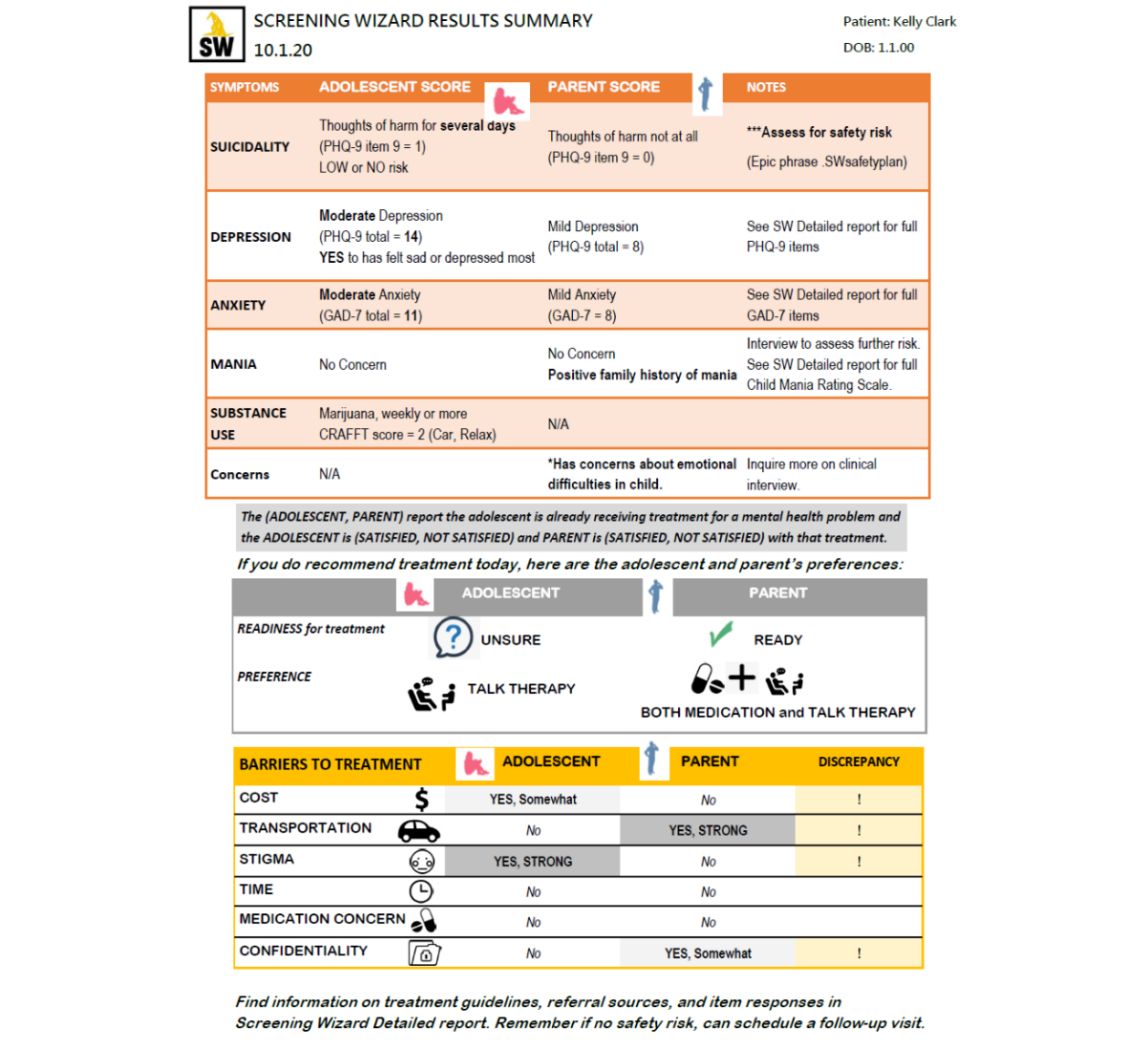
Mock-up of the Screening Wizard report for providers. CRAFFT: Car, Relax, Alone, Forget, Friends, Trouble; GAD-7: Generalized Anxiety Disorder-7; N/A: not applicable; PHQ-9: Patient Health Questionnaire-9; SW: Screening Wizard.

The initial SW prototype was developed iteratively by the first and last authors, starting with low-fidelity mock-ups (Figures 2 and 3). Content was selected for each domain by choosing measures that are *free, brief, and validated* to maximize the likelihood of uptake [31]. Measures with as few items as possible, but with adequate reliability and validity, were selected from the public domain or made available at no cost (Table 1). The initial algorithms were iteratively refined through expert consultations. The concept and initial design were based on an earlier decision support tool designed to balance symptom severity with child and parent preferences when making treatment recommendations for childhood anxiety [32].

**Table 1:** Screening Wizard constructs and measures (total time=7 minutes).

Construct	Measure or instrument	Adolescent version	Parent version (reporting on their child’s symptoms)	Time per construct (minutes)
Depression severity	PHQ-9^a^ (11-item adolescent version, 9-item parent about adolescent) [33]	✓^b^	✓	1
Suicidality	Y-CAT^c^ Static Suicidality [34] (12 items); item 9 on PHQ-9	✓	✓^d^	1
Anxiety	GAD-7^e^ (8 items) [35,36]	✓	✓	1
Mania	CMRS^f^ brief [37] (12 items), family history	✓	✓	1
Substance use screen	Past year frequency of substance use (tobacco, alcohol, marijuana, prescription drugs not prescribed to them, illegal drugs, inhalants, and herbs or synthetic drugs; 7 items) and CRAFFT^g^ [38] (6 items)	✓	N/A^h^	1
Concern about impairment or functioning	Single item (concern about child’s social or emotional functioning)	N/A	✓	<1
Treatment history and satisfaction	Currently in treatment? Satisfied with your care? (2 items)	✓	✓	<1
Readiness for treatment	Single item about readiness to start treatment if recommended that day	✓	✓	<1
Treatment preferences	Single item (medication, talk therapy, both, no preference, or not interested in treatment)	✓	✓	<1
Potential barriers	Cost, time, stigma, confidentiality, medication side effect concern, and transportation (6 items) adapted from abbreviated measure of Barriers To Adolescents Seeking Help Scale [39,40], Parental Barriers to Help Seeking Scale [41], and Antidepressant Meaning Scale [42]	✓	✓	<1

^a^PHQ-9: Patient Health Questionnaire-9.

^b^Construct present.

^c^Y-CAT: Youth-Computerized Adaptive Screen (Static Version).

^d^Only item 9.

^e^GAD-7: Generalized Anxiety Disorder-7.

^f^CMRS: Child Mania Rating Scale.

^g^CRAFFT: Car, Relax, Alone, Forget, Friends, Trouble.

^h^N/A: not applicable.

### Study Design

#### Participants

To elicit feedback from the populations that would ultimately use SW, we conducted interviews with adolescents who had a history of depression and their parents (parents and adolescents were interviewed separately). In addition, we conducted focus groups with health care providers. These providers were included if they were part of the day-to-day clinical staff, which could include physicians, advanced practice practitioners (Certified Registered Nurse Practitioners and physician assistants), nurses, patient care technicians, patient service representatives, embedded mental health therapists, or administrative staff such as practice managers at a pediatric practice. The practices chosen were participating in a pediatric practice-based research network, Pediatric PittNet, that was considering (but had not yet begun) implementing SW as part of a proposed trial to evaluate SW compared with treatment-as-usual. These providers, as well as adolescents and parents who were recruited, were located in the state of Pennsylvania. We conducted interviews with parents and adolescents to gain their individual perspectives and to ensure their confidentiality as they talked about sensitive mental health topics. We conducted focus groups with providers within the same practice to benefit from group interactions in response to questions about practice-level experiences and opinions on SW implementation. Interviews and focus groups were conducted until the study principal investigators felt that they had sufficient information to move forward with implementation.

#### Recruitment

Participants were recruited through primary care clinics and flyers posted in clinical settings; clinics were selected for flyering based on the study teams’ previous experience with those clinics and confidence in those clinics’ depression diagnosis. Interested individuals could self-refer through these means. Research assistants also received referrals from the same clinical settings and approached families after they expressed interest in participating in the research. Eligible families were included if the child was aged between 12 and 17 years and screened positive for depressive symptoms or suicidal behavior at their pediatric primary care office. Individuals were excluded if they did not undergo screening in a pediatric primary care setting and were not referred as a result of a positive depression or suicide screening. Health care providers needed to be employed in a pediatric primary care setting to participate in the focus groups.

#### Procedure

At a scheduled visit occurring in the research offices, parents and youth were provided with a verbal consent script, and research assistants reviewed it before completing the interviews. Health care provider focus groups took place during a scheduled break at the pediatric primary care offices, and all providers underwent a verbal informed consent process. Interviews and focus groups were conducted by a member of the research team with deep knowledge of SW and previous experience conducting qualitative interviews (BGM). Data collection was performed by a qualitative methodologist (MH). All interviews and focus groups were audio recorded and transcribed.

#### Interview Guide

All participants were asked to describe previous experiences with screening for depression in primary care, following which they were shown a mock-up of the SW tool and asked for their feedback on it. In the first part of the interview, both adolescents and parents were asked if they or their child had ever been screened before; details of where, when, and how that screening occurred; what the results of the screening had been and how results indicating depression were handled; and whether and how they had been referred to mental health services as a result of the screening. They were also asked whether and how depression screening should occur in primary care. In the focus groups, the first portion of the focus group included questions about how depression screening is currently done in their practice as well as how positive screenings and referrals are handled. In the second part of the interviews and focus groups, participants were shown a mock-up of the SW provider decision tool paper report. The interviewer explained each component of the report and provided a low-fidelity version of the SW tool through Adobe software on an iPad (as it would be administered in a pediatric primary care setting). Participants responded with their reactions and thoughts on the appropriateness of the types and amount of information included in the provider decision tool. Participants explained out loud using a think-aloud approach [43] as they completed the low-fidelity SW tool. They were asked to review what they thought each question meant and how relevant the questions would be for their health care provider to provide advice on mental health treatment. The interview and focus group guides are included in Multimedia Appendix 1.

#### Data Analysis

Interviews and focus groups were transcribed verbatim, and codebooks were inductively developed based on the content. In total, 2 qualitative analysts (BGM and MH) independently coded 10 interview transcripts to establish intercoder reliability. Cohen κ scores were calculated for each code; the average score was 0.76, indicating substantial agreement [44]. The remaining 12 interview transcripts were coded by the primary coder. Both focus group transcripts were cocoded, and coding disagreements were adjudicated to full agreement. Completed coding was used to produce thematic analyses of the interviews and focus groups [45]. The resulting themes were shared with and agreed upon by the secondary coder and interviewers as a form of investigator triangulation. All study procedures were approved by the institutional review board of the University of Pittsburgh.

## Results

### Overview

Demographics of the sample are summarized in Table 2.

**Table 2:** Participant demographics.

Demographic characteristics			Adolescents (n=11)		Parents (n=11)		Health care providers (n=18)^a^
**Age (years)**							
	Value, mean (SD)	15 (0.6)		41 (8.2)		45 (12.8)	
	Value, range	15-17		32-61		28-69	
**Sex at birth, n (%)**							
	Female	5 (45)		9 (82)		15 (83)	
	Male	6 (55)		2 (18)		2 (11)	
**Provider type, n (%)**							
	Physician	N/A^b^		N/A		4 (22)	
	Certified registered nurse practitioner	N/A		N/A		1 (6)	
	Administrator	N/A		N/A		2 (11)	
	Professional staff nurse or nurse coordinator	N/A		N/A		5 (28)	
	Patient care technician	N/A		N/A		1 (6)	
	Patient services representative (front desk staff)	N/A		N/A		3 (17)	
	Behavioral health therapist	N/A		N/A		1 (6)	

^a^One health care provider has missing data, as they only partially participated in the focus group because of clinical care needs and did not provide demographic data.

^b^N/A: not applicable.

We conducted interviews with 11 parents and 11 adolescents and conducted two focus groups with 18 health care providers (physicians, Certified Registered Nurse Practitioners, nurses, behavioral health providers, and support staff) at 2 pediatric practices participating in a pediatric practice-based research network, Pediatric PittNet, which were in the process of considering implementing SW as part of a proposed trial to evaluate SW compared with treatment-as-usual. We identified five main themes across the interviews and focus groups: (1) parents, adolescents, and pediatric PCPs and support staff believe that depression screening should occur in pediatric primary care; (2) there is concern that accurate self-disclosure does not always occur during depression screening; (3) SW is viewed as a tool that could facilitate depression screening and that might encourage more honesty in screening responses; (4) parents, adolescents, and providers do not want SW to replace mental health discussions with providers; and (5) providers want to maintain autonomy in treatment decisions. Each of these themes will be discussed below with select quotations from participants presented in Textbox 1.

**Selected quotes from adolescents, parents, and providers.**

**Themes and Quotes**

Parents, adolescents, and pediatric primary care providers and support staff believe that depression screening should occur in pediatric primary care

- Q1. “A lot of the time maybe people are suffering from mental health problems may not want to talk about it outright and may not bring it up, so if they give you like a survey or something that might help kind of encourage them to talk about it more.” [adolescent]
- Q2. “I mean catching it earlier on is better than catching it when [...] they’ve already suffered for 4 or 5 years, you know, [with] kind of underlying issues building up, getting worse.” [parent]
- Q3. “It would just seem more helpful and useful to like know what the person goes through before you try to do something. You know what I mean?” [adolescent]
- Q4. “I think that [primary care] should be where it should come from. I mean that’s where we go when we’re sick, that’s where we go for guidance.” [parent]
- Q5. “I feel like it’s easier to talk to a doctor about it rather than talking to like your family [...] because it seems—they’ve dealt with it before, they kind of already know, they’ve seen it, it’s easier for them to understand it rather than someone who doesn’t have any experience with it.” [adolescent]
- Q6. “Once you start screening then the doctor—if the doctor posits a question and the children will like yeah, so it’s a double edge sword on that. Um—I think if there’s—if they have any types of symptoms maybe they should [screen] but other than that I-I would say uh—no I guess.” [parent]
- Q7. “[the PCP] is supposed to help me out with like with something like if like—if I have like a broken leg or something.” [adolescent]

There is concern that accurate self-disclosure does not always occur during depression screening

- Q8. “I just don’t have the confidence that they understand the questions. [...] So we have to navigate that you know: ‘[Do] you understand why we’re asking this? Do you understand what these questions are?’, and you know I feel like there’s probably kids that slipped through the cracks that don’t screen positive that would probably benefit from a more intensive review, and that puts us in a difficult position ‘cause ‘Oh yeah, your screen is negative’ but we’re in the room and we know it’s a chaotic situation or we can tell [from] the kids affect or from other you know just red flags. That always scares me, and you feel like you’re gonna miss something.” [provider]
- Q9. “I wasn’t completely honest when I first filled out like the tablet [...] because I didn’t want to get treatment. [...] Like if they asked me if my mood was like 1 through 10, say it was like a 1 I said it was like a 3 or 4.” [adolescent]
- Q10. “fear of it getting out, like if their parents were to find out. I know that it’s harder to open up if you think more people are going to know about it ‘cause it feels like people are judging you.” [adolescent on why adolescents may be dishonest in screening]

Screening Wizard is viewed as a tool that could facilitate depression screening and that might encourage more honesty in screening responses

- Q11. “When you’re talking to a person, you’re thinking about what they’re thinking, but when you’re talking to a computer, you know it’s not, like, judging you or having any thoughts of its own. It’s just recording what you’re inputting.” [adolescent]
- Q12. “I think having both the teen and the parent do them separately of course um has the benefit of getting a more accurate picture for the same reasons, like the teenager might be embarrassed to admit uh sort of things but the parent will have noticed things the they’ll be honest about it, vice versa the parent might be embarrassed to admit that their teenager has some problems but the teenager might be willing to admit it um and so having these two perspectives you’re just a lot more likely to actually catch something that needs attention.” [parent]
- Q13. “So that [discrepancies between parent and child] is useful to know about beforehand so I can address it, and address it in a way that is immediate. So, [pretends they are talking to a patient] ‘Let me show you the handout and, this is what you guys told me.’ I don’t know makes it more natural to just bring it up in conversation.” [provider]

Parents, adolescents, and providers do not want the Screening Wizard to replace mental health discussions with providers

- Q14. “I wouldn’t want it [Screening Wizard] to replace dialogue but obviously you know a screen that’s positive or issues will lead to dialogue but a negative screen doesn’t mean there’s no issues you know? So I think that it can’t really replace dialogue. [Even if the screening is negative] I think that the provider can follow up with dialogue that just says you know the-the screening that you did um seems like you guys are doing really well which is pretty unusual for teenagers...” [parent]
- Q15. “I think you’re delegating a lot of important conversations to, just the use of handouts and these screener questionnaires and then yeah it is depersonalized. But if you kind of use it in a way that prompts important conversations, then it doesn’t have to be [depersonalized].” [provider]
- Q16. “[Screening Wizard should be used] when you go in for like a checkup or something, see like um how the person’s feeling and if they are feeling bad maybe you can um talk to them about it during the checkup or something like that.” [adolescent]

Providers want to maintain autonomy in treatment decisions

- Q17. “If you give me a recommendation it might work the vast majority of the time but I think a lot of us especially with gray hair are going to say my clinical judgment would probably trump the decision support rarely but just enough that I don’t want you to take it away, you know I’m going to use it but I want that final veto power.” [provider]
- Q18. “There might be situations where I don’t always agree with the decision support and now I’m like ‘Well, that’s not really what I wanna do’ and-and that, that makes me a little less comfortable.” [provider]
- Q19. “How are we supposed to reconcile the difference between the decision support that could spit out versus the parent who really wants something else [...] ‘cause I can tell you that it’s going to happen and I just, I with our-our population I know that’s going to happen.” [provider]
- Q20. “If a tool like this gives me decisions support, makes me feel like I’m making the right decision that it is okay to wait then I’m really glad about that. But on the other hand if your decision support sets the bar so low because of medical legal fear issues that everyone has to be referred, right now if I don’t get that referral done now you’re making me feel like I’m at risk, you know not only for the kid which of course is the primary issue but now medically, legally you’re telling me I should do something I don’t do it if something bad happens and that’s on the chart I feel like some lawyers going to find that and I’m now you know, ‘Why didn’t you do that?’ So the standard of care, best support kind of decision making really has to be very carefully thought out because you know it’s obviously the goal is to keep the kid safe but at the same time you’ve got to protect us a little. If you tell me to do something and I don’t do it am I liable? I’m worried about that, which is I’m going to be honest.” [provider]

### Theme 1: Parents, Adolescents, and Pediatric PCPs and Support Staff Believe That Depression Screening Should Occur in Pediatric Primary Care

Parents and adolescents were nearly unanimous in believing that universal depression screening should occur and that pediatric primary care was an appropriate place for it. Depression screening was regarded as appropriate because it might identify adolescents struggling with depression who, for any number of reasons, might not know that they were experiencing depression or who might not be comfortable bringing the topic up unless asked. Adolescents, in particular, thought that they and their peers would be unlikely to bring it up on their own but might disclose if asked (Q1; Textbox 1). Some adolescents and parents regarded the PCP as a more neutral person to talk to about depressive symptoms than a parent. The possibility of identifying depression earlier, as well, was regarded as a benefit by all groups of participants (Q2 and Q3; Textbox 1). In addition to depression screening itself being a good thing, pediatric primary care was regarded as a good place to do it (Q4 and Q5; Textbox 1).

Health care providers were also in favor of routine depression screening in primary care, and the PCPs who participated in the two focus groups described routine depression screening as part of their practice. In one case, a health care provider described their experience as a parent, as well, who wished that the practice they took their child to would universally screen for depression. Providers’ reasoning for why screening should take place coincided with the reasoning described by adolescents and parents in terms of the importance of detecting mental health problems early and PCPs being able to help families with follow-up.

Only 1 parent and 1 adolescent (their child) felt that universal depression screening should not occur in primary care. The parent felt that pediatricians should screen for depression in the presence of symptoms, but that to do so otherwise might somehow draw an adolescent’s attention to depression such that they might become depressed when they were not before (Q6; Textbox 1). The adolescent did not think that their pediatrician should handle mental health concerns and instead thought that depression screening was the purview of a therapist (Q7; Textbox 1).

### Theme 2: There Is Concern That Accurate Self-disclosure Does Not Always Occur During Depression Screening

Although pediatricians were in favor of and described routinely conducting depression screening, they noted that they did not treat screening results as accurate, meaning they often perceived a high rate of false-negative results for screening. A positive depression screen was likely to be regarded as accurate and acted upon with referral to mental health services or prescription of antidepressants (depending on patients’ and PCPs’ comfort and preference), but a negative screening did not indicate to pediatricians that there was no cause for concern (Q8; Textbox 1). One pediatrician described checking in with his patients using open-ended questions even following a negative screening to try to prevent patients who actually have depressive symptoms from being missed.

Adolescents and parents were additionally concerned that adolescents might not answer screening questions honestly. Some adolescents described not filling out depression screenings accurately in the past out of a desire to avoid treatment (Q9; Textbox 1). In addition to a desire to avoid treatment, adolescents sometimes described not wanting to be honest in screening for fear of others finding out that they have mental health problems (Q10; Textbox 1). Parents also expressed concern that their children might lie during screenings, either exaggerating or downplaying how they were really feeling out of a desire for *attention* or because they were in denial about symptoms. Thus, although screening was valued, it was not regarded as perfect in its ability to identify adolescents with depression.

### Theme 3: SW Is Viewed as a Tool That Could Facilitate Depression Screening and That Might Encourage More Honesty in Screening Responses

When presented with the SW tool, nearly all interviewees and focus group participants had a positive reaction to the screening tool. One adolescent had a negative reaction but indicated that he would not have wanted to do any screening, and so his sentiment was around screening in general, not SW in particular. The fact that the screening was delivered via tablet was regarded as a major selling point by adolescents and by their parents reporting on what adolescents would like. Parents noted that their children were extremely comfortable interacting with the technology. Both parents and adolescents felt that the lack of a paper hardcopy that could be misplaced made them feel more secure in the confidentiality of their responses. They were aware that the provider would receive a printout of results, but this did not impair their perception of confidentiality. Adolescents sometimes described feeling more comfortable disclosing depression in a computerized screening (Q11; Textbox 1), and parents loved the idea of filling out their own evaluation of their child’s depressive state on the tablet (Q12; Textbox 1). Furthermore, one primary point of difference in opinion between parents and adolescents in these interviews was the extent to which the parents should be involved in their child’s screening, with parents expressing a greater desire to participate and be present for it than children desiring to have their parents present for their screening. Giving the parents an opportunity to fill out the screening as well was regarded by them as helpful and giving them a voice in their child’s care.

Providers in the focus groups were comfortable with the administration of screening on a tablet and supportive of other features of SW (resource sheets for adolescents and families, the screening of parents, and feedback to clinicians regarding whether parents and children are on the same page with regard to symptoms and treatment). They were particularly supportive of seeing both parent and child screening responses and of seeing discrepancies between the parent and child responses highlighted on the SW report. One provider described how they would use such a report to guide discussion with the parent and child (Q13; Textbox 1).

### Theme 4: Parents, Adolescents, and Providers Do Not Want SW to Replace Mental Health Discussions With Providers

As noted previously in theme 2, depression screening was not regarded as always accurate, and as such, despite strongly positive feedback on SW, parents, adolescents, or providers did not want SW to replace face-to-face discussions of screening results and mental health between providers and patients. One parent shared that they envisioned a provider following up on a negative screening by having a more open-ended conversation that confirmed (or perhaps refuted) the original negative screening (Q14; Textbox 1). Adolescents and providers also often envisioned SW as operating best in a context in which the screening is followed up with a conversation with a provider (Q15 and Q16; Textbox 1). As long as SW was a tool that helped facilitate these sorts of discussions rather than something that replaced them, participants felt that it would prove beneficial.

### Theme 5: Providers Want to Maintain Autonomy in Treatment Decisions

In addition, providers expressed concern about the idea of SW making treatment recommendations. Providers were concerned that they might think a recommendation should not be followed (Q17; Textbox 1), contributed to by the concern for false-negative results based on an adolescent’s willingness to be honest on the screen. Although they were personally interested in seeing recommendations so that, as one provider put it, “old dogs could be shown new tricks,” they were opposed to those recommendations being either requirements (as part of an SW trial studying efficacy; Q18; Textbox 1) or shown to parents and adolescents via an SW output sheet. Their concerns about recommendations being shared with patients stemmed from wanting to avoid conflict if they disagreed with the recommendation (Q19; Textbox 1). Providers also expressed concern about avoiding legal liability in the event that they did not follow a recommendation and there was a bad outcome (Q20; Textbox 1).

## Discussion

### Principal Findings

In this qualitative study, we elicited feedback from parents, adolescents, and health care providers in pediatric primary care regarding the process of screening adolescents for depression and suicidality and their opinion about a technology-based decision support tool—SW—aimed at guiding PCPs in responding to positive depression and suicidality screens in adolescents. Although most groups felt that depression screening should occur in pediatric primary care, the most salient concerns raised were around nondisclosure on the screen, which may impede the identification of depressed or suicidal adolescents. Respondents felt that the SW tool could elicit more honest responses than routine screening and that it adds value of contrasting parent and adolescent barriers and preferences, but they did not want SW to replace mental health discussions or treatment decision-making.

The benefits of depression screening in primary care were seen in identifying symptoms earlier, comfort with the pediatric PCP, and disclosure to a safe supportive adult such as the PCP being more likely than the adolescent’s parent. This aligns with formative literature on depression screening in pediatric primary care, which found that parents, adolescents, and PCPs find depression screening acceptable [46]. This literature contributed to informing the United States Preventive Services Task Force guidelines [47] as well as guidance from organizations such as the American Academy of Pediatrics [48,49] to recommend routine depression screening. Interestingly, 1 parent and their child expressed concern that depression screening may lead to depression in the child and that screening should be done by a mental health professional. Although clinicians are generally familiar with the literature that asking about suicide and self-harm does not lead to an increase in these behaviors [50], parents and adolescents may be less familiar, warranting the addition of reassurance within the introduction to a screen. Similar to this dyad, some parents and adolescents may have the opinion that their PCP office should not provide mental health care because of a lack of expertise. Although pediatric PCP offices continue to expand depression screening, some may lack messaging that reduces stigma and enhances confidence in their capacity to manage mental health concerns. Including antistigma messaging, such as that promoted by the Healthcare Equality Index [51], for sexual and gender minorities with regard to mental health within a PCP office’s marketing, signage, and within the screen itself may help increase parent and adolescent comfort with screening and enhance trust in the PCP practices’ competence to address mental health concerns.

All participant groups shared a concern that adolescents may not be honest during the screening process. In studies evaluating the validity of the PHQ-9 in the adolescent population, Richardson et al [33] found a 10% false-negative rate in the screen when compared with the Diagnostic Interview Schedule for Children Version IV *gold standard* diagnostic tool. As this study was conducted in a research population, actual clinical populations may have even higher false-negative rates because of active nondisclosure to their clinician, which may occur because of the perceived consequences of disclosure. Adolescents may worry that the PCP will share sensitive answers or results with their parents, be unwilling or embarrassed to discuss symptoms with their PCP, or be unwilling to engage in treatment and therefore obscure symptoms. A 2014 systematic review found that screens administered in pediatric primary care are more likely to be seen as acceptable when there are clear statements made to adolescents and parents that the screens are confidential and being applied universally [52]. Although the rates of screen positivity for depression in pediatric primary care are approximately 12% [33,53], approximately one-third (32%) of high school students say they have felt sad or hopeless every day for 2 weeks or more in the past year on the 2019 Youth Risk Behavior Surveillance System survey [1], which is anonymous, thereby increasing the likelihood of honesty. Although one factor contributing to lower positivity on screens may be because of universal screening occurring during well-child visits, which adolescents with depression are less likely to present for compared with acute or emergency visits [54-56], another modifiable factor is a lack of honesty.

Adolescents, parents, and providers held the opinion that the SW tool may encourage more honesty and thereby facilitate depression screening. This is partially because of it being an electronic screen [57], as adolescents shared that they would feel less judged by a computer, and it is something they are already comfortable interacting with. A study asking 115 adolescents about their comfort with screening found that only 70 (60.9%) agreed that they were honest on paper screens compared with 102 (88.7%) who were honest when completing electronic screens [57]. In addition, providers thought there would be less of a chance of missing positive symptoms if both parents and adolescents completed the screen, as opposed to just adolescents alone, as is commonly practiced. The GLAD-PC guidelines recommend soliciting information from both adolescents and parents independently [48]. Providers may not be aware of the discrepancies between adolescents and parents regarding mental health treatment preferences or barriers to treatment [10,19]. If parents and adolescents disagree on the presence of symptoms, adolescents may be less likely to receive care [58,59]. SW specifically highlights symptoms, preferences, and barriers from both adolescent and parent perspectives, as in Figure 1, thereby offering an opportunity for providers to facilitate SDM between all parties, which may lead to increased uptake and engagement with the treatment decision.

Although adolescents, parents, and providers saw value in SW, they did not want the technology to replace discussions about mental health or management decisions. Although providers anticipated that SW may enhance honest symptom reporting by the adolescent, they also hesitated to rely completely on the screen if they had clinical suspicion for mental illness not revealed by the screen. Other studies have shown that PCPs tend to not use screens as intended, using individual items to inform treatment as opposed to severity symptom scores [7]. In addition, because pediatric PCP comfort varies in managing mental health diagnoses [60] and the ability to follow recommendations is dependent on the setting and available resources, providers may feel a one-size-fits-all PCP management approach is not warranted. Although the treatment decision algorithm was a major component of the initial SW tool, we concluded that the providers’, adolescents’, and parents’ discomfort with this portion of the report may preclude future implementation. Nonetheless, measurement-based care may improve depression outcomes [61] and is facilitated by computerized decision support, which may enhance compliance with clinical guidelines [62] and result in small to moderate improvements in quality of care [63]. Future iterations of SW will, instead of providing specific treatment recommendations (eg, refer for substance use treatment or follow-up in 6 weeks), gather and display information in a user-friendly way to the provider while highlighting the severity score of symptoms and discrepancies between adolescents and parents and providing reference to the current clinical guidelines to enhance clinical decision-making without being overly prescriptive.

### Limitations

This provider sample was recruited from a well-resourced setting with regard to access to mental health services and is likely not representative of all pediatric primary care settings. For our purposes, we were interested in developing a technology to assist PCPs in mental health care management. Therefore, a sample having some familiarity with the mental health care system was helpful in facilitating discussions. Future iterations of SW will likely need to be adapted for other settings, but we feel that most potential acceptability concerns were identified in this process. The adolescent age range was small, and much younger adolescents and older young adults may have different opinions about the SW tool. Another limitation is that we did not collect information about gender or sexual minority status, which is important because of the elevated risk of depression, or about individually reported race or ethnicity. These important demographic data will be collected in future interviews. Owing to the limitations of this study population, we do not feel that thematic saturation was reached regarding all possible responses to or opinions on SW. However, the opinions expressed in the interviews and focus groups were saturated for the purposes of this study population—that is, with the exception of one parent-child dyad with responses that differed from the others, responses were consistent across the interviews and in the two focus groups. Additional interviews with respondents from different settings may have yielded different opinions or themes.

### Conclusions

By providing the PCP with (1) both adolescents’ reports and parents’ reports of depression, (2) reports of potential mental health and substance use comorbidities, (3) an additional screen for suicidality alone, (4) both adolescents’ and parents’ treatment preferences, (5) adolescents’ and parents’ potential barriers to treatment, and (6) an overview of discrepancies between the adolescents and parents, our aim is for SW to address the potential gaps in routine screening and facilitate the SDM process between adolescents, parents, and PCPs. In this formative study evaluating the initial design of SW, we learned that providers, parents, and adolescents are concerned that there are limitations to the current efforts to routinely screen adolescents for depression, mainly because screens may miss some adolescents who are unwilling to disclose symptoms. They recognized value in SW as a computerized tool may elicit more honest responses and that asking both adolescents and parents about symptoms and summarizing discrepancies may be useful. None of the groups wanted the tool to replace treatment discussions, and providers did not want it to replace their clinical judgment.

### Future Implications

Although SW was originally developed as a treatment decision algorithm, this qualitative study informed us to remove this component and instead focus on aspects identified as most useful by all groups: identifying discrepancies between adolescents and parents and efficiently presenting treatment barriers and preferences to health care providers. This formative work guides the iterative development of the SW tool, which will then be evaluated in a future effectiveness trial to understand whether it facilitates SDM and enhances treatment uptake for adolescent mental health concerns in the primary care office.

## Appendix

Multimedia Appendix 1 Screening Wizard interview guide.

## Abbreviations

GLAD-PC: Guidelines for Adolescent Depression in Primary Care
PCP: primary care provider
PHQ: Patient Health Questionnaire
SDM: shared decision-making
SW: Screening Wizard
