# Supplementary material for: Perceived Facilitators of and Barriers to Implementation of a Decision Support Tool for Adolescent Depression and Suicidality Screening: Focus Group and Interview Study
Source: JMIR Ment Health. 2021 Sep 15;8(9):e26035. doi: 10.2196/26035 (PMC8482166; doi:10.2196/26035)
Supplement: Multimedia Appendix 1 [file mental_v8i9e26035_app1.docx]

**Provider** Interview/Focus Group Questions

The first questions are about your general practices surrounding screening and treatment referral for depression and suicidality among adolescents and any perceptions surrounding this practice.

1. **What is your current practice after you have someone who screens positive for depression?**

***…How do you interpret the screen? Do you categorize the number based on severity? Do you use additional assessment tools? What other questions do you ask? How do you introduce treatment options? How do you involve the parent?***

1. **Do you prescribe antidepressants yourselves?**

***…Would receiving guidance on when to prescribe antidepressants and which antidepressants to prescribe be helpful to you?***

*Display PowerPoint or handout with more detailed description of proposed Screening Wizard (SW) intervention, an automated, decision-support system that will provide PCPs with information and facilitate formal procedures to follow-up on positive test as recommended by the USPSTF. We will describe how we envision this intervention working and what information it would provide. Provide examples of different potential iterations of what the output of SW could look like including original handout, a longer multi-page printout, or a picture of an online clinician portal where you would see the original handout but be able to click through for more individual item scores, reference of the scale, its sensitivity/specificity, etc.*

1. **Do you think this intervention will disrupt or support your workflow?**

***…Do you think adolescents and parents will complete the tablet screener? Would you have time to get a printout from a printer? Would you have time to look at the printout? Would there be a better way to get you this information?***

1. **What are your thoughts about the output provided to you from the Screening Wizard? [show example]**

***…Is the format useful? Is it too much/too little information? How would the output change or inform your management? How do you see yourself using it? Does it have the right information you need (can re-review the survey questions)? How would you imagine yourself using it? Would you think you would rather have answers to individual questions or a summary of just the screening results and advice on how to act on the results? Do you feel like you could trust the results if they were summative versus individual item?***

1. ***What other information do you feel like it would be helpful to know in addition to the screen***

***results?***

1. **We are considering providing you also with personalized education materials you could use to address any of the concerns raised by the screen (e.g. stigma resources, parent guidance re: mental health insurance). How do you envision yourself using this? Would you want an accompanying script? [provide examples of potential handout with or without script]**
2. **What, if anything, would be the benefits of this Screening Wizard – to you, to your patients/their parents, to your practice?**
3. **What, if anything, might get in the way of Screening Wizard and act as barriers - to you, to your patients/their parents, to the practice?**
4. **Who in your practice would be most influential to determining policies and procedures that would pertain to implementation of Screening Wizard?**
5. **Before we end, is there anything else you’d like to share?**

**Parent** Interview

First, I’d like to ask some questions about your experiences with care your child has received with their primary care doctor, or other providers within that practice.

1. **Do you think that screening for depression is something that primary care doctors should do?**
2. **To your knowledge, has your child ever received screening for depression at their doctor’s office?**

**If yes:**

- 1. **Can you tell me about what that processed entailed? At what point in the visit did the screening occur? How was the screening done (electronically, on paper, with a person)?**
  2. **Were you involved in this screening? Do you think you should have been involved in the screening? Why or why not?**
  3. **How do you feel about completing a screener about your child?**
  4. **Did your child’s doctor discuss the screening and your child’s answers with you or your child? If yes, how did that discussion go?**
  5. **What about this process did you find helpful/unhelpful?**

**If no:**

**a. If your child’s doctor were to screen your child for depression, how do you think this process should occur? Should you be involved in the screening? Why or why not? If you think you should be involved, how should you be involved? How do you feel about completing a screener about your child?**

1. **Has your child ever received a referral for therapy, medication, or another type of mental health treatment from their primary care doctor?**

**If yes:**

- 1. **Can you tell me more about the referral? How was that referral discussed with you? How was it discussed your child?**
  2. **Did you feel your doctor took your preferences into account in making the referral? How did the doctor consider your perspective, if at all?**
  3. **Did you feel your child’s preferences were taken into account in making the referral? How did the doctor consider your child’s perspective, if at all?**
  4. **What about this process did you find helpful/unhelpful? How could it be improved?**

**If no:**

- 1. **If your child’s doctor were to make a mental health referral for your child, what do you think would be the most important things they should consider?**

1. **When there is a disagreement between a parent, child, and a doctor about a mental health referral, what should happen? How should a decision be made about what to do next?**
   1. **If needed: If a parent and a child want a mental health referral, but the PCP doesn’t think it is necessary, what should happen?**
   2. **If needed: If the PCP wants to refer a child for mental health care, but the child and parent don’t want it, what should happen?**
   3. **If needed: If the PCP wants to refer a child for mental health care, and the child also wants the referral, but a parent does not, what should happen?**

*PowerPoint or handout with more detailed description of proposed Screening Wizard intervention, an automated, decision-support system that will provide PCPs with information and facilitate formal procedures to follow-up on positive screen for depression and/or suicidality in youth. We will describe how we envision this intervention working and what information it would provide.*

1. **What are your initial thoughts toward the Screening Wizard intervention?**
2. **What are your thoughts on the aesthetic design including: colors, instructions, format of each part of the Screening Wizard intervention? Were any parts of navigating through questions on the iPad confusing, e.g. you didn’t know where to go next? What improvements would you make?**
3. **What general questions or concerns do you have when hearing about the idea of this screening tool?**
4. **What are your concerns about confidentiality and safety of the tool?**
5. **Can you think of other types of questions or topics that should be asked of yourself or your child on the screen? What are the most important topics to ask about on the screen?**
6. **What, if any, health benefits do you think might come from using Screening Wizard?**
7. **Is this what you would expect in an intervention to better identify adolescent depression and suicidality? What’s missing?**

**Are there any other comments or questions you would like to share?**

**Youth** Interview

First, I’d like to ask some questions about your experiences with care you’ve received with your primary care doctor (pediatrician), or other providers within that practice.

1. **Do you think your doctor should ask you about your mental health, such as screening for depression (define e.g. surveys to teens to ask questions about symptoms that may suggest they have depression or thoughts of suicide).**
2. **Have you ever received screening for depression at your doctor’s office?**

**If yes:**

- 1. **Can you tell me about what that processed entailed? At what point in the visit did the screening occur? How was the screening done (electronically, on paper, with a person)?**
  2. **How were you involved in the screening-i.e., did you answer questions? Did you parent answer them? Did you parent see your answers?**
  3. **Did your doctor discuss the screening and your answers with you?**
  4. **What about the screening did you find helpful/unhelpful?**

**If no:**

**a. How should you be screened for depression? Who should take part in the screening-you by yourself? You and your parents? Only your parents? (Interviewed should probe for why they are answering as they are, if they don’t volunteer the reason.)**

1. **Has your doctor referred you to mental health care? Has your doctor suggested you take medication for your mental or emotional health, talk to a counselor, or suggest another type of mental health care?**

**If yes:**

- 1. **Can you tell me more about that experience? How was the referral discussed with you? With your parent?**
  2. **Did your doctor take into account your view when they referred you? How did the doctor consider your view, if at all?**
  3. **Did your doctor take your parent’s view into account when they referred you? How did the doctor consider your parent’s view, if at all?**
  4. **What about this process did you find helpful/unhelpful?**

**If no:**

- 1. **If your doctor were to make a mental health referral for you, what do you think would be the most important factors that they should consider?**

1. **When there is a disagreement between a parent, child, and a doctor about a mental health referral, what should happen? How should a decision be made about what to do next?**
   1. **If needed: If a parent and a child want a mental health referral, but the PCP doesn’t think it is necessary, what should happen?**
   2. **If needed: If the PCP wants to refer a child for mental health care, but the child and parent don’t want it, what should happen?**
   3. **If needed: If the PCP wants to refer a child for mental health care, and the child also wants the referral, but a parent does not, what should happen?**

*PowerPoint or handout with more detailed description of proposed Screening Wizard intervention, an automated, decision-support system that will provide doctors with information and help provide ways to follow-up with youth who say they are depressed or suicidal. We will describe how we envision this intervention working and what information it would provide.*

1. **Imagine if you arrived to your doctor’s office and received an iPad, which included text asking if you’d like to participate in Screening Wizard. What would you think of this?**
2. **What are your thoughts on the colors, instructions, and format or display of each part of the Screening Wizard? Were any parts of navigating through questions on the iPad confusing or unclear, e.g., you didn’t know where to go next?**
3. **Can you describe when you think this screening should be use (i.e. in the doctor’s office waiting room, in the exam room? With parents present or not?) Is there anything that you think might make a teen less likely to take this screening, or less likely to answer the screening questions honestly?**
4. **What any general questions or concerns you have when hearing about the idea of this enhanced screening tool?**
5. **We think your parent would fill out a questionnaire, and “opt in” to a study about screening wizard and then you would also fill out the questionnaire. What you fill out should be private, but we are thinking about making a handout for your parent. This handout might give the results of the screen-for example, it could say you are moderately depressed and have mild anxiety but are not suicidal. Would you feel comfortable with your parent having this information? If yes, why and in what format? If no, why and how much if any information would you feel comfortable with your parent having? Should teens filling out Screening Wizard, be able to check off what their parent’s info handout should look like (e.g. include or not include my depression score)?**
6. **What are your concerns about privacy of your information and safety of the screening?**
7. **Can you think of other types of questions or topics that should be asked of teens on the screen?**
8. **What, if any, health benefits do you think might come from using Screening Wizard?**
9. **Is this what you would expect in a screening to better identify teens who are depressed and might be thinking of suicide? What’s missing?**

**Are there any other comments or questions you would like to share?**
